# Population screening for glucose-6-phosphate dehydrogenase deficiencies in Isabel Province, Solomon Islands, using a modified enzyme assay on filter paper dried bloodspots

**DOI:** 10.1186/1475-2875-9-223

**Published:** 2010-08-05

**Authors:** Melissa Kuwahata, Rushika Wijesinghe, Mei-Fong Ho, Anita Pelecanos, Albino Bobogare, Losi Landry, Hugo Bugora, Andrew Vallely, James McCarthy

**Affiliations:** 1 Queensland Institute of Medical Research, Herston, QLD, 4006, Australia; 2 Pacific Malaria Initiative Support Centre, Australian Centre for International and Tropical Health, School of Population Health, University of Queensland, Herston, QLD, 4006, Australia; 3 National Vector Borne Disease Control Program, Ministry of Health, Honiara, Solomon Islands; 4 School of Medicine, University of Queensland, Herston, QLD, 4006, Australia

## Abstract

**Background:**

Glucose-6-phosphate dehydrogenase deficiency poses a significant impediment to primaquine use for the elimination of liver stage infection with *Plasmodium vivax *and for gametocyte clearance, because of the risk of life-threatening haemolytic anaemia that can occur in G6PD deficient patients. Although a range of methods for screening G6PD deficiency have been described, almost all require skilled personnel, expensive laboratory equipment, freshly collected blood, and are time consuming; factors that render them unsuitable for mass-screening purposes.

**Methods:**

A published WST8/1-methoxy PMS method was adapted to assay G6PD activity in a 96-well format using dried blood spots, and used it to undertake population screening within a malaria survey undertaken in Isabel Province, Solomon Islands. The assay results were compared to a biochemical test and a recently marketed rapid diagnostic test.

**Results:**

Comparative testing with biochemical and rapid diagnostic test indicated that results obtained by filter paper assay were accurate providing that blood spots were assayed within 5 days when stored at ambient temperature and 10 days when stored at 4 degrees. Screening of 8541 people from 41 villages in Isabel Province, Solomon Islands revealed the prevalence of G6PD deficiency as defined by enzyme activity < 30% of normal control was 20.3% and a prevalence of severe deficiency that would predispose to primaquine-induced hemolysis (WHO Class I-II) of 6.9%.

**Conclusions:**

The assay enabled simple and quick semi-quantitative population screening in a malaria-endemic region. The study indicated a high prevalence of G6PD deficiency in Isabel Province and highlights the critical need to consider G6PD deficiency in the context of *P. vivax *malaria elimination strategies in Solomon Islands, particularly in light of the potential role of primaquine mass drug administration.

## Background

Glucose-6-phosphaste dehydrogenase (G6PD) is an important enzyme in cellular metabolism in the first and rate-limiting step of pentose-phosphate pathway. Among the functions of this pathway is the protection of cells from oxidative stress, through its role in conversion of NADP to NADPH, thereby replenishing the levels of reduced glutathione. As erythrocytes lack other detoxifying enzymes, people with G6PD deficiency are susceptible to oxidative stress in their red blood cells. The G6PD gene is located on the X chromosome, and as a result deficiencies show X-linked inheritance, whereby a higher proportion of males suffers from the deficiency. A wide array of genetic variants has been identified, which confer differing phenotypes. These include the well characterised G6PDA^- ^and G6PD Mediterranean variants [1,2].

An estimated 400 million people carry a deficient variant of the G6PD gene, with disproportionately higher prevalence observed in tropical regions [1-3]. The fact that the prevalence of G6PD deficiency correlates with endemicity for malaria had lead to the hypotheses that G6PD deficiency may be result of natural selection conferring protection against malaria infection [2-5]. However, studies have indicated that this protection may only occurs with certain G6PD variants, and that differing level of protection are likely to be seen in hemizygous males, homozygous and heterozygous females [1,4,6-8].

With the exception of rare sporadic WHO class I individuals, most G6PD deficient individuals are asymptomatic [1,2]. However, specific triggers such as ingestion of certain foods (e.g. fava beans), exposure to oxidative drugs, and infection can cause haemolytic anaemia, and in severe cases haemolysis sufficiently severe to require blood transfusion. Such adverse events are generally confined to individuals with G6PD activity < 10% of normal (WHO Class II).

The anti-malarial drugs primaquine, dapsone, and the experimental drug tafenoquine are oxidative anti-malarials and can cause haemolytic anaemia. Primaquine is the most important of these, being the only approved anti-malarial that can be used to eliminate *Plasmodium vivax *hypnozoites and *Plasmodium falciparum *gametocytes. As a result there is increasing public health concern with implementation of malaria elimination initiative worldwide using mass drug treatment programmes [9-11]. Studies of tolerance of primaquine undertaken in G6PD deficient individuals have suggested that a course of lower dosage treatment such as 0.75 mg/kg/week for eight weeks or 15 mg/day for 14 days compared with the standard 30 mg/day for 14 days may be safe [10,12,13]. However, given the wide genetic and phenotypic variability of G6PD, it is considered a priority to undertake phenotypic and genetic analysis, and perhaps a carefully controlled tolerance trial on different populations where primaquine MDA is being considered for elimination [14,15].

Direct testing of the enzymatic activity of G6PD on a freshly collected blood sample is the most widely used diagnostic method for diagnosis of deficiency. Methods used include older tests such as brilliant cresyl blue decolourization test and methaemoglobin reduction test [16,17]. The methodology recommended by the International Committee for Standardisation in Haematology is the NADPH fluorescent spot test, which requires a UV lamp [18,19]. These method all have shortcomings that limit their use in mass-screening or in field settings [18]. Other methods that do not require UV lamp have been used for screening studies include the ring method and Sephadex gel MTT-PMS method [20-24]. These methods are also used as a diagnostic test prior to primaquine treatment in larger hospitals and health centres in developing countries, where the necessary facilities and equipment are available. Other metods that have been described for tesitng include cytochemical assays [25], and DNA sequence analysis of the G6PD gene. The former has the advantage of being a reliable method for detection of hemizygous deficient males, homozygous deficient females, or heterozygous deficient females because the G6PD status of individual erythrocytes is tested [26]. DNA sequence analysis reqiures analysis of the whole gene which spans 18kB of genomic sequence [1]. However, all of these tests suffer from limitations that inhibit their utility for in-field mass-screening purposes, due to factors such as technical expertise required, expense, duration of test procedure, sensitivity of reagents to light and heat, low detection threshold, or relatively low throughput capacity [22,24].

A new enzymatic method was described by Tantular and Kawamoto that utilizes a terazolium salt known as WST8, and a less light-sensitive form of the PMS hydrogen carrier, called 1-methoxy PMS [27]. The reduced light sensitivity and the colourimetric nature (both qualitative and quantitative) of this method, and the possibility of using it with dried bloodspot make it potentially more suitable for field use [28]. In this manuscript a method for screening for mass screening for G6PD deficiencey is described. It is modified from the WST8 method and was optimizied to a 96-well plate format and using dried blood spots with internal standards as controls. The present study reports the evaluation of this method for in-field mass-screening of G6PD to determine the prevalence of G6PD deficiency in Isabel Province, Solomon Island.

## Methods

### Population and sample collection

This study took place in October 2009 as part of a comprehensive baseline malaria survey conducted to inform the implementation of the national malaria control and elimination programme in Solomon Islands. The survey was undertaken by the Vector Borne Disease Control Programme (VBDCP) in collaboration with the Australian government funded Pacific Malaria Initiative Support Centre (PacMISC; a Brisbane-based consortium between the School of Population Health at the University of Queensland; the Queensland Institute of Medical Research; and the Australian Army Malaria Institute). The survey was undertaken in Isabel Province, which consists of a main island, Santa Isabel, and many other smaller surrounding islands. A total of 41 villages in Isabel Province, were visited over the course of 28 days. Samples were collected, after informed consent, from 8541 males and females of all ages. Isabel is predominantly populated with people of Melanesian ethnicity and therefore specific ethnicity data was not collected.

Blood spots were collected from finger-prick blood onto 3 MM filter paper (Whatman) and were dried at ambient temperature. The dried blood spots were stored individually in zip-lock bags containing silica desiccant beads and refrigerated (4-8°C) whenever possible. All samples were assayed within 10 days from collection.

### Assay mixes and controls

A concentrated assay mix was prepared beforehand in Australia and frozen aliquots transported to Isabel Province. The mix contained 50 mM glucose-6-phosphate (Oriental Yeast Co. Japan), 4 mM NADP (Merck Pty Ltd.), 1 M Tris-HCl pH7.2-7.5 and 100 mM MgCl_2 _(Sigma-Aldrich). A second mix was also prepared to serve as substrate negative control. This control mix lacked glucose-6-phosphate and NADP. WST-8 -1-methoxyPMS was obtained from Dojindo Laboratoreis (Japan). Working assay mix was prepared just prior to setting up each assay. For each 96-well plate 0.5 mL of concentrated mix and 0.5 mL of WST8 reagent were added to 19 mL of water. A no-substrate control mix was also prepared with 1 mL water, 26.3 μL concentrated no-substrate mix, and 26.3 μL WST8 reagent.

To calibrate the assay panel of reference control samples with defined G6PD activity were prepared. A commercial standard reagent of known normal G6PD activity (G6PD control Normal, Trinity Biotech) that consists of lyophylised G6PD in human red cell hemolysate base with stabilisers and preservatives [29] was used to create a panel of internal controls that were used in each assay. These consisted of normal, moderate (WHO Class III deficient ~30% enzyme activity), and severe (WHO Class II deficient ~10% enzyme activity) deficiency, and no enzyme (0%) control. To prepare moderate and severe controls, the reference normal G6PD control sample was diluted with human blood that had been rendered to have no G6PD activity by heating to 50°C for 20 minutes; for the no-enzyme control only the inactivated blood was used. Aliquots of these control samples were frozen until required. G6PD activity was classified using a slightly modified version of a previously published reference range [2], < 10% activity (WHO Class I-II) was classified as severe deficiency, while < 30% enzyme activity was classified as moderate deficiency.

### G6PD assay

On each day when the assays were undertaken a set of controls (normal, moderately deficient, severely deficient, no-enzyme) were taken and spotted onto 3 MM filter paper. A single ^1^/_16 _inch diameter disc (FISKARS Hand Punch) was punched out from each blood-spot sample, and placed in a single well of a 96-well flat bottom microplate (Greiner Bio-One). Control spots and an extra bloodspot for no-substrate control were also punched and placed in allocated wells. One well remained empty and served as a "no sample" negative control. 200 μL of working assay was then pipetted and mixed into each well except the substrate negative control well, into which 200 μL of no-substrate assay mix was added. Plates were then incubated for 2 hours at ambient temperature.

**Figure 1:**
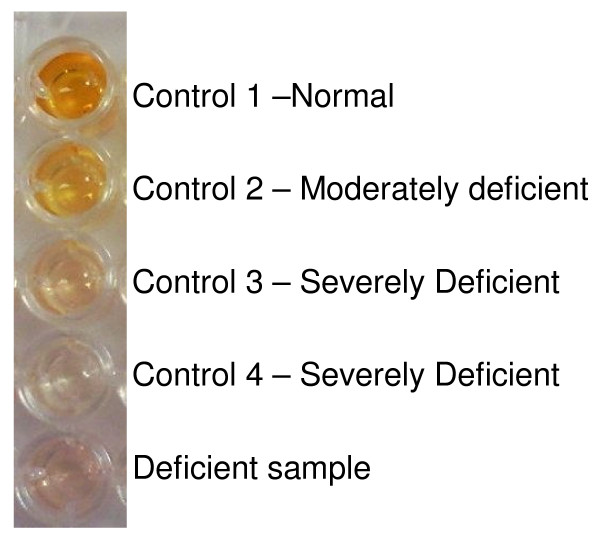
**Appearance of assay plate wells for testing of G6PD activity from dried blood spots**. The distinct yellow colour (first well) indicates a sample with normal G6PD activity, the well with pale yellow colour (second well) represents a sample with moderate deficiency, and the remaining three almost colourless wells are indicative of severe deficiency.

96 well plates were first inspected by eye, and then absorbance quantified in a microplate reader (Chromate 4300, Awareness Technology) at OD_450-595 _. Normal G6PD activity is indicated by dark yellow to orange colouration, while severe and moderate deficiency appearing as almost colourless, and faintly yellow colouration respectively (Figure 1). Although for the purpose of this study only deficient (severe or moderate)/normal status were recorded, it was possible to distinguish intermediate from normal activity by their intermediate yellow colouration and absorbance.

### Preliminary experiments

To determine limitations of the storage condition of bloodspots and assay mixes two separate pilot experiments were undertaken prior to the field survey. The storage stability of G6PD enzyme as bloodspots were assessed at 4°C, 25°C and 37°C over a period of two weeks using bloodspots with known G6PD activity. Bloodspots used in the experiment were manually blotted blood containing differing amount of commercial normal G6PD control (5%, 10%, 15%, 25%, 50%, complete normal). These spots were dried and stored in zip-lock bags containing silica gel until tested. In a separate preliminary experiment the performance of stored assay mixes was also evaluated. Assay mixes were prepared as a complete mix of all required reagents, then stored at ambient temperature in the dark for 4 days, 1 week, 2 weeks, 3 weeks and 4 weeks, and were used to assay control bloodspots.

### Comparison of WST8 screening assay with other assays for G6PD

To evaluate the WST8 assay as a screening method, blood was collected by venipuncture into EDTA vacutainers from 20 individuals at Buala Hospital in Isabel Isalnd. These blood samples were tested using the WST8 assay, an FDA-approved G6PD rapid diagnostic test (BinaxNow, Inverness Medical), and by quantitative biochemical testing using a commercial assay kit (Trinity Biotech) at the Pathology department of Royal Brisbane and Women Hospital, Brisbane, Australia within four days of collection. To control for possible loss of enzyme activity in collected blood stored until testing, blood samples from two investigators with known levels of G6PD activity were collected at the same time as the clinical sample.

### Ethics

Ethical approval to undertake the malaria survey including the G6PD prevalence survey was obtained from the Solomon Islands Health and Ethics Committee; the Research Ethics Committees of the University of Queensland and the Queensland Institute of Medical Research; and from the Australian Defence Human Research Ethics Committee.

### Statistical analysis

Chi-square and odds ratio were used to test for any significant differences in prevalence of G6PD deficiency between males and females. Geographical Information System (GIS) data, including the village point information, was used in ArcGIS software (ESRI) to assess for spatial autocorrelation using Moran's I statistics. Moran's I value of +1 represents correlation, -1 represents dispersion and 0 indicates a random spatial pattern. Spatial cluster analysis using SaTScan (v7.0.3) was also performed to explore for evidence of clustering of G6PD deficiency. Standardized residuals were calculated using chi-square analysis to assess for differences in prevalence of G6PD deficiency between villages to identify villages that may have shown an unexpectedly higher proportion of moderate deficiency.

## Results

### Evaluation of bloodspot and assay mix storage

Bloodspots stored at 4°C, 25°C and 37°C were assayed on Day 1, 5, 10 and 14 to determine its effect on enzyme degradation and the resulting reduced G6PD activity. The Day 1 assay indicated that only bloodspots with known enzyme activity of < 10% tested deficient, with no differences apparent between blood spots stored at different temperatures. (Table 1). However, the test results at Day 5 indicated that only those bloodspots stored at 4°C were equivalent to those obtained on Day 1. At 25°C and 37°C those with original activity of 15% were being judged as deficient (< 10%), indicating that ≥ 5% of activity was lost. As storage duration and temperature increased, bloodspots with original activity of 15-50% tested deficient in the assay indicating ≥ 5-40% loss of activity. Our experiment showed reduced activity of ~5% in bloodspots stored for 10 days at 4°C, and for 5 days at 25°C and 37°C, a finding that indicates that bloodspots could be stored within these limitations and still yield reliable results.

**Table 1:** The effect of storage temperature and duration on dried blood spot estimation of GPD enzyme activity

Storage temperature	Baseline G6PD enzyme activity (%) that results in a sample testing severely deficient (< 10% activity) at the designated storage temperature and duration				Decay in enzyme activity
	**Day 1**	**Day 5**	**Day 10**	**Day 14**	

**4°C**	10%	10%	15%	15%	~ 5% by Day 10
**25°C**	10%	15%	25%	25%	~ 5% by Day 5
					~ 15% by Day 10
**37°C**	10%	15%	25%	50%	~ 5% by Day 5
					~ 15% by Day 10
					~ 40% by Day 14

The reliability of G6PD assays on blood spots stored for different times at different temperatures was assessed with bloodspots containing differing amount of reference G6PD enzyme activity. The table shows the % enzyme activity that tested as deficient (≤ 10% activity) at days 1, 5, 10 and 14 when stored at 4°C, 25°C and 37°C. The decay in enzyme activity represents the difference between the original enzyme activity and the activity when retested.

**Figure 2:**
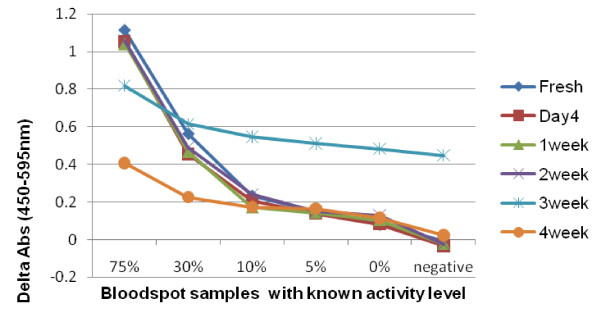
**Assay performance with assay mix stored for up to 4 weeks**. Absorbance readings from bloodspot samples with known levels of G6PD using assay mixes stored for varying durations at ambient temperature. Each line represents assay results with a different assay mix. Uniform assay performance was observed with assay mixes stored for up to 2 weeks, but abnormal or degrading performance was observed in those stored for 3 and 4 weeks.

The stability of assay mixes was likewise evaluated (Figure 2). Assay mix stored up to two weeks at ambient temperature showed relatively stable assay performance compared to freshly prepared assay mix. Assay mix stored for three weeks or more showed reduced or aberrant performance.

### Assay validation

Testing was undertaken on 20 individuals at Buala Hospital using three methods: the WST8 assay, the FDA-approved RDT BinaxNow, and a quantitative enzymatic assay undertaken at the Pathology department, Royal Brisbane and Women's Hospital in Australia a few days later. Table 2 displays the results from the three tests. Of the 20 individuals, all three tests indicated that 14 people showed normal activity. Definitive enzyme testing and WST8 assay identified the same five deficient individuals and one with intermediate activity. The BinaxNow RDT identified the same six individuals as deficient, but was unable to distinguish the individual with intermediate enzyme activity from the deficient individuals.

**Table 2:** Comparison of G6PD test results using different assay methods

Number	Gender	WST8	RDT (BinaxNow)	RBWH Clinical Pathology laboratory
1	M	Deficient	Deficient	0.6
2	M	Deficient	Deficient	0.6
3	M	Deficient	Deficient	0.6
4	M	Deficient	Deficient	0.3
5	M	Deficient	Deficient	0.5
**6**	**F**	**Intermediate**	**Deficient**	**7.9**
7	F	Normal	Normal	13.8
8	F	Normal	Normal	12.9
9	F	Normal	Normal	13.6
10	M	Normal	Normal	13.4
11	F	Normal	Normal	13.6
12	F	Normal	Normal	14.3
13	M	Normal	Normal	12.9
14	F	Normal	Normal	12.2
15	F	Normal	Normal	13.4
16	F	Normal	Normal	12.7
17	M	Normal	Normal	15.6
18	M	Normal	Normal	15
19	M	Normal	Normal	10
20	F	Normal	Normal	13.2
Control 1	M	Normal	Normal	15.2
Control 2	F	Normal	Normal	12.8

Pathology testing 9-20 U/gHb is classified as Normal.

### G6PD deficiency prevalence

**Figure 3:**
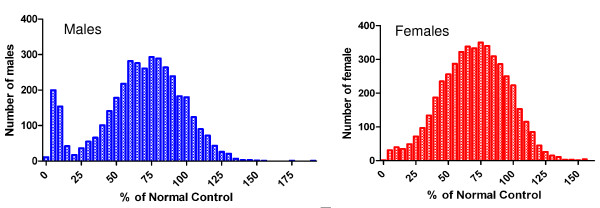
**Distribution of G6PD activity in Isabel Island by gender**. OD measures were converted to % activity relative to normal control and were graphed to visualise any difference in distribution pattern of G6PD activity between males and females.

Results from 8,541 individuals were analysed, comprising 3,880 males and 4,636 females. The G6PD deficiency prevalence is summarized in Table 3. Analysis revealed that overall 1,731 individuals (20.3%) showed G6PD deficiency, and of these 590 individuals (6.9%) were observed to have severe deficiency (< 10% of normal enzyme activity). While there was only a slight preponderance of overall deficiency among males, with deficiency of 21.7% (n = 843) and 19.1% (n = 886) respectively for males and females, there was a significantly higher prevalence of severe deficiency among males compared to females, 10.9% (n = 421) and 3.6% (n = 168) respectively (p < 0.0001, OR = 3.2, 95% CI 2.7-3.9). This gender specific difference is apparent on inspection of a graphical representation of the distribution of activity percentage relative to normal G6PD activity (Figure 3).

**Table 3:** G6PD deficiency prevalence in Isabel Province and prevalence by gender (n(%))

	Total (%)	Male (%)	Female (%)
**Samples tested**	8,541	3,880 (45.6)	4,636 (54.4)
**Normal**	6,810 (79.7)	3,037 (89.9)	3,750 (80.9)
**Total deficient^†^**	1,731(20.3)	843 (21.7)	886 (19.1)
**Severe deficient^††^**	590 (6.9)	421 (10.9)	168 (3.6)
**Moderate deficient^†††^**	1,141 (13.4)	422 (10.9)	717 (15.5)

† < 30% normal enzyme activity

†† < 10% normal enzyme activity

††† 10-30% normal enzyme activity

### Prevalence by village

**Figure 4:**
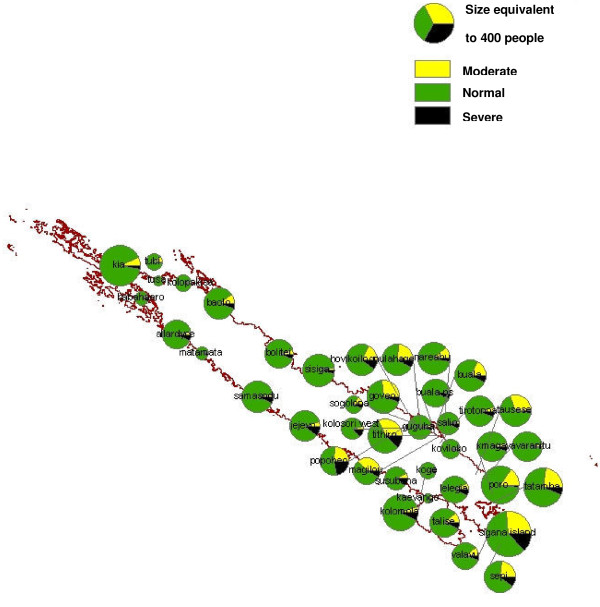
**Map of G6PD deficiency prevalence in Isabel Province**. G6PD deficiency prevalence in villages was mapped to compare prevalence among villages. High severe deficiency (> 10%) were observed in Susubona, Jejevo, Hovikoilo, Kolosori West, Sigana, Popoheo and Thithiro.

G6PD deficiency was mapped using GIS data obtained during the survey and from the 1999 census by the Solomon Island Ministry of Lands, Housing and Survey (Figure 4). Prevalence of ≥ 10% severe deficiency was observed in Jejevo, Susubona, Hovikoilo and Kolosori West, Sigana, Popoheo, Thithiro. Although severe deficiency was not observed in Kolopakisa or Tusa, the number of people sampled from these villages was small.

**Figure 5:**
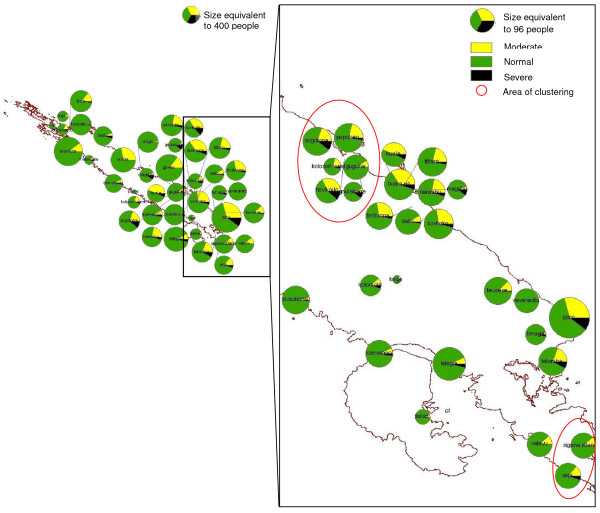
**G6PD deficiency in Females in Isabel Province**. Spatial cluster analysis with SaTScan revealed clustering of villages with higher G6PD severe deficiency prevalence among Females (p = 0.001). Map shows prevalence distribution in Isabel province.

The village-wise G6PD deficiency prevalence data was assessed for spatial autocorrelation using Moran's I statistics, and for spatial cluster using SaTScan (v7.0.3). Moran's I values obtained for severe deficiency in females, males and both genders combined were close to 0.00 and p values were greater than 0.10 (Table 4). This indicated that prevalence of severe G6PD deficiency is neither correlated nor dispersed, and that it is randomly distributed across Isabel Province. Spatial cluster analysis scan was undertaken to identify clusters with high rates of severe deficiency using Poisson model. This analysis identified two clusters for severe G6PD deficiency in females. The first cluster was located on the south-eastern area consisting of Hovikoilo, Kolosori West, Sogolona, and Popoheo (p = 0.001), and the second cluster included Sepi and Sigana (p = 0.001) which are located on the southern tip of Isabel Island (Figure 5).

**Table 4:** Spatial autocorrelation (Moran's I statistics) of village-wise severe G6PD deficiency

	Females	Males	Combined
**Moran's I**	0.18	0.32	0.19
**Z score**	0.32	0.55	0.35
**P**	> 0.10	> 0.10	> 0.10

## Discussion

Various screening methodologies used to assay G6PD activity require laborious techniques and equipment such as shakers, heated water baths, UV-lamps, spectrophotometers, microscopes or DNA sequencing apparatus. Without significant financial, infrastructural, and technical support many of these methods are not suitable for field-based mass-screening as is likely to be required to support primaquine mass drug administration, implemented as part of a malaria elimination programme. To develop an assay more suitable for this purpose, we have adapted an existing published methodology for mass-screening utilizing dried filter paper bloodspots on a 96-well microtitre plate which enables assaying G6PD of large numbers of blood spots in places with limited laboratory facilities. To improve assay robustness we developed a range of controls suitable for calibration of results in a field setting.

Evaluation of the performance of this semi-quantitative G6PD assay using blood from 20 individuals from Buala village in Isabel Province, Solomon Islands against an FDA-approved G6PD RDT and quantitative biochemical testing indicated high level of agreement, and confirmed the ability of this G6PD assay to identify deficient individuals. Interestingly, it was also possible to identify the female subject who showed mild deficiency (most likely a heterozygote) using the assay. However, confirming her status would require supplemental testing such as a cytochemical assay [26].

This G6PD deficiency study took place in conjunction with a malaria survey which already required sampling of finger-prick blood for microscopy and filter paper for PCR. Use of finger-prick blood has been found to be more acceptable in various cultures and communities around the world, and allows for less-laborious collection of large number of samples [30,31]. Additionally, blood collected on filter paper can be stored in bulk and processed. The G6PD deficiency survey therefore was easily integrated into the broader malaria survey. Future malaria surveys that are conducted to monitor transmissions could include this assay at little overall additional cost and resource requirement.

This study has indicated that G6PD deficiency is present in Isabel Province, with a prevalence of severe deficiency of 6.9%. As expected, the prevalence of severe deficiency was significantly higher in males (10.9%) than in females (3.6%) (p < 0.0001). Although the prevalence of severe deficiency was prominently higher in males, when looking at moderate deficiency alone, slightly higher prevalence was found in females than males (15.5%, vs. 10.9%). This could be due to a predominating G6PD variant that phenotypically expresses a severe form of deficiency resulting in large number of heterozygous females but fewer males present with moderate activity of G6PD. Heterozygous females are difficult to identify using enzymatic tests, as they can have vastly different enzyme levels depending on the variant and level of mosaicism [32,33]. It is not possible to distinguish between the homozygous and heterozygous females in the population or to determine the G6PD variant without undertaking cytochemical or molecular analysis [7,34].

The overall prevalence of severe deficiency of 6.9% (10.9% in males) is similar to prevalence levels reported in other studies that have been undertaken in the Southwest Pacific, such as in Vanuatu (6.8% - 7.4%) [5,35]. An earlier study conducted in Solomon Islands included a small group of people from Isabel Province, and found a deficiency rate of 17-24.1%, including moderate deficiencies [36]. Our study also showed an average prevalence of 20.27% (21.73% in males) including moderate deficiencies. Studies in areas of Papua New Guinea and New Britain have reported prevalence of G6PD deficiency ranging from 0-53% and 0.8-23% respectively [37,38]. In the study reported by Gorman and Kidman [37], the variation in prevalence was found to correlate with the altitude, suggesting a role of selective advantage due to protection from malaria as transmission follows similar patterns [37]. Furthermore, different linguistic groups from the same regions showed different prevalence of G6PD deficiency. Studies from Papua New Guinea suggest that separate and distinct evolution of red blood cell traits exist due to combination of selective advantage and relative isolation of populations [38-40]. Considering the existence of the very large number of linguistic groups, and that many of the islands present dramatic topographic barriers, differences in intra- and inter-island prevalence in Vanuatu and Solomon Islands could be expected.

While several villages were identified as having severe deficiency prevalence of over 10%, spatial autocorrelation of village-wise prevalence data from Isabel Province showed that there is no spatial pattern of neither correlation or dispersion of severe deficiency prevalence. Village-wise prevalence of G6PD severe deficiency showed a random spatial pattern. However, spatial cluster analysis with SaTScan revealed two clustering regions of G6PD severe deficiency prevalence in females. Although clustering of deficiency in females may imply that some consanguinity may exist within these villages, the overall spatial analysis data maybe suggesting that there has been ample gene flow across villages (inter-village marriages) or that there has not been isolated exposure of confined selection pressure needed to establish distinct patterns of G6PD deficiency prevalence in Isabel Province. However, further investigation is needed to determine the true cause. Malaria transmission history and genotyping of the G6PD gene from this population may be able to shed light on this.

In several villages a significantly higher total prevalence of G6PD deficiency was observed (30-45%). However, whether this represents a higher prevalence of deficiency or is due to other factors such as suboptimal assay performance in very wet conditions cannot be determined without repeating the tests in these villages. Bloodspots were collected from these villages on rainy and particularly humid days, when it was observed that blood spots did not saturate through the filter paper, but rather smeared across a larger surface area. It is possible that this effect was due to filter papers becoming damp before blood samples were taken. The consequence of using such bloodspots is that the volume of blood in each punched spot is lower than a completely saturated bloodspot, thereby impeding comparison against the controls. Furthermore, samples collected on such days were also difficult to dry. Drying is an important step, especially for the storage and consistent elution for the assay [30,41]. Both of these considerations may have confounded measurement of G6PD activity, and have led to a greater proportion of individuals showing lower normal activity or moderate deficiency. To minimize these issues, it is recommended that filter paper be stored in zip-lock bags containing silica gel desiccants before being used for blood collection, and perhaps change of silica gel desiccants frequently after collection for samples that were not able to be dried sufficiently due to wet weather conditions.

In this respect, the red cell volume is known to affect the outcome of assays for G6PD activity [42]. Formal testing of G6PD activity is accompanied by a haematocrit/haemoglobin testing for this reason. The effect of differing levels of haematocrit on this assay was not explored in this study, and it may represent a factor that requires consideration in future studies.

Aside from the potential problems discussed above the assay performed well, with the majority of samples (n = 8,541) collected and processed in 28 days by just by 1 or 2 operators. In this survey, storage conditions were not constrained and assay mixes and reagents were able to be stored at optimal storage temperatures. However, in preliminary experiments it was observed that prepared mixes can be used up to two weeks when stored at ambient temperature in the dark. This may allow assays to be done during a two-week period in isolated field settings where access to refrigerator/freezer is limited. The preliminary experiments also showed that bloodspots can be stored for up to 10 days if stored at 4°C or up to 5 days at ambient temperature with only about 5% reduction in activity. Therefore, it would be possible to undertake batch processing if there is a suitable number of operators to process large number of samples at a time.

## Conclusions

With the progress of worldwide efforts to eliminate malaria, G6PD deficiency and mass drug administration with primaquine has assumed increasing importance to countries affected by *Plasmodium vivax *infection. If implemented safely and effectively, MDA could be a powerful strategy as a step towards elimination [11,43]. To assess the prospect of primaquine MDA the prevalence of G6PD deficiencies in areas of interest must be estimated. In this study, the applicability of a filter paper mass-screening assay to provide prevalence of G6PD deficiency in Isabel Province was evaluated. This study showed that it was possible to undertake a large-scale mass screening of G6PD deficiency in the field using the filter paper assay in order to assess prevalence of the deficiency. Results of the survey indicate that G6PD deficiency is prevalent in Isabel Province, and as a consequence this must be taken into consideration with any future planning of strategies for malaria elimination in this Province.

## Competing interests

The authors declare that they have no competing interests.

## Authors' contributions

MK participated in the survey, carried out the G6PD assay experiments and testing, undertook the analysis and drafted the manuscript. RW facilitated and supported the survey and MFH contributed to the assay experiments, AP undertook mapping analysis. AB, LL and HB participated in its design and supervised field coordination. AV and JM conceived the study and contributed in its design. All authors read and approved the final manuscript.
